# Pentagalloyl Glucose: A Review of Anticancer Properties, Molecular Targets, Mechanisms of Action, Pharmacokinetics, and Safety Profile

**DOI:** 10.3390/molecules28124856

**Published:** 2023-06-19

**Authors:** Chengli Wen, Nathupakorn Dechsupa, Zehui Yu, Xu Zhang, Sicheng Liang, Xianying Lei, Tao Xu, Xiaolan Gao, Qinxue Hu, Phattarawadee Innuan, Jiraporn Kantapan, Muhan Lü

**Affiliations:** 1 Department of Intensive Care Medicine, Affiliated Hospital of Southwest Medical University, Luzhou 646000, China; wenchengli076@swmu.edu.cn (C.W.); leixianying310@swmu.edu.cn (X.L.); daishaoye2239@sina.com (T.X.); gaoxiaolan656@swmu.edu.cn (X.G.); 15893082457@163.com (Q.H.); 2 Molecular Imaging and Therapy Research Unit, Department of Radiologic Technology, Faculty of Associated Medical Sciences, Chiang Mai University, Chiang Mai 50200, Thailand; nathupakorn.d@cmu.ac.th (N.D.); phattarawadee_in@cmu.ac.th (P.I.); 3 Luzhou Key Laboratory of Human Microecology and Precision Diagnosis and Treatment, Luzhou 646000, China; zhangxu290823@163.com (X.Z.); liangpharm@swum.edu.cn (S.L.); 4 Laboratory Animal Center, Southwest Medical University, Luzhou 646000, China; yuzehui_swmu@outlook.com; 5 Department of Gastroenterology, Affiliated Hospital of Southwest Medical University, Luzhou 646000, China

**Keywords:** pentagalloyl glucose, gallotannin, anticancer, molecular targets, mechanisms, pharmacokinetics, safety profile

## Abstract

Pentagalloyl glucose (PGG) is a natural hydrolyzable gallotannin abundant in various plants and herbs. It has a broad range of biological activities, specifically anticancer activities, and numerous molecular targets. Despite multiple studies available on the pharmacological action of PGG, the molecular mechanisms underlying the anticancer effects of PGG are unclear. Here, we have critically reviewed the natural sources of PGG, its anticancer properties, and underlying mechanisms of action. We found that multiple natural sources of PGG are available, and the existing production technology is sufficient to produce large quantities of the required product. Three plants (or their parts) with maximum PGG content were Rhus chinensis Mill, Bouea macrophylla seed, and Mangifera indica kernel. PGG acts on multiple molecular targets and signaling pathways associated with the hallmarks of cancer to inhibit growth, angiogenesis, and metastasis of several cancers. Moreover, PGG can enhance the efficacy of chemotherapy and radiotherapy by modulating various cancer-associated pathways. Therefore, PGG can be used for treating different human cancers; nevertheless, the data on the pharmacokinetics and safety profile of PGG are limited, and further studies are essential to define the clinical use of PGG in cancer therapies.

## 1. Introduction

Cancer is a significant public health concern worldwide. Despite the advances in early diagnostic strategies and targeted therapies, the incidence of cancer continues to increase with high mortality [1]. Currently, numerous cancer treatments are available, including surgery, chemotherapy, hormonal therapy, radiation, immunotherapy, and targeted therapies. Chemotherapy is the most common systemic cancer treatment, and chemotherapeutic drugs damage DNA to kill the cells or inhibit their growth. However, the treatment has several adverse effects, including hematological toxicity, disrupted gastrointestinal activity, alopecia, altered neurological activity, anaphylaxis, hepatotoxicity, and nephrotoxicity [2]. Therefore, plant-derived natural compounds with excellent safety profiles and minimal toxicity are ideal alternatives for safe and effective single or combined cancer therapies [3].

Natural products are the sources of complex molecules for discovering new drugs or lead compounds [4]. Natural compounds and their derivatives have long been used for treating many diseases [5,6], and their anticancer properties are an active research area. Apart from their antioxidant and anti-inflammatory activities, natural products also modulate multiple signal transduction pathways associated with cell survival, proliferation, differentiation, migration, angiogenesis, hormone activities, detoxification, and immune responses. In addition, several authors have documented the anticancer effects of natural polyphenols [3,7,8,9].

**Figure 1:**
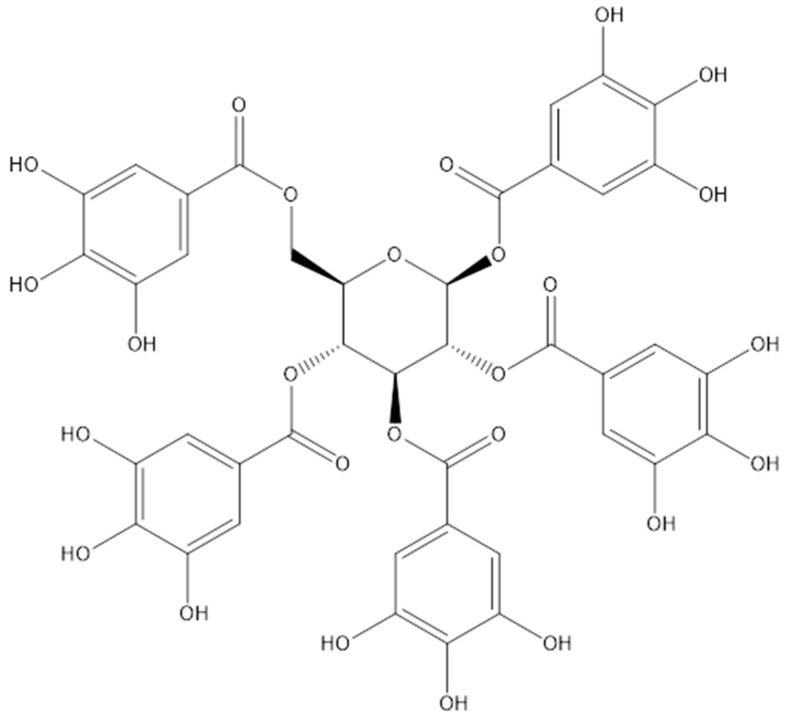
Chemical structure of the pentagalloyl glucose.

Penta-O-galloyl-D-glucose, also known as pentagalloyl glucose (PGG), is classified as a gallotannin; it is a hydrolyzable tannin abundant in many plants, including Rhus chinensis, Paeonia suffruticosa, Bouea macrophylla, and Toona sinensis [10,11,12,13]. The naturally occurring polyphenolic compound PGG exists in beta-PGG form, whereas an anomeric alpha-PGG is rarely found in nature [14]. However, both compounds can be chemically synthesized, and a highly purified material is obtained after crystallization [15]. Gallotannins are composed of gallic acid molecules bound to a central D-glucose via ester bonds (Figure 1). It can exist either in free form or as a core structure of tannic acid. PGG is mainly found in plants as a precursor of high molecular weight compounds, namely galloyl glucose, gallotannins, and ellagitannins [16], which impart astringency to the plant [17]. Notably, PGG has higher hydrophobicity than other polyphenols (octanol/water partition ratio of PGG: 129, gallic acid: 7.76, and hexagalloyl glucose: 1.51) [18]. Compared with rigid 1,2-di-O-galloyl-4,6-valoneoyl-β-d-glucose molecules, PGG is flexible with a lower surface area and higher dipole moment [19]. Moreover, PGG has potent biological activities, including antioxidative, anti-inflammatory, antiviral, antimicrobial, antidiabetic, and anticancer. In addition, PGG can reduce abdominal aortic aneurysms [20,21]. Notably, the anticancer properties of PGG have great clinical value and need further exploration. Therefore, this review focused on the anticancer activity of the purified PGG-enriched crude extracts and the mechanism of action involved.

## 2. Sources of PGG

### 2.1. Synthesis

PGG can be synthesized biologically and chemically, and the processes have been extensively reviewed by Torres-León et al. [22]. The biosynthesis of PGG comprises the consecutive esterification of gallic acid and glucose [23], whereas the chemical synthesis of PGG involves the methanolization of tannic acid in an acetate buffer [24]. 

### 2.2. Natural Sources

Several plants are the natural sources of PGG; however, the concentration varies among different genera and species. The maximum extraction yield of PGG was from gallnuts (58.40 g/kg) [25], followed by that from the *B. macrophylla* seeds (52.88 g/kg) [12] and the Mangifera indica kernels (50.03 g/kg) [26]. Multiple parts of a plant contain PGG. For example, PGG is found in Punica granatum leaves, seeds, fruits, and peel. In contrast, it is found in the young and old leaves, peel, kernel, and bark of *M. indica*. In addition, PGG concentrations are different in different parts of a plant. For instance, the yields of PGG from *M. indica* young leaves, old leaves, peel, kernel, and bark were 23.26, 1.82, 17.71, 50.03, and 0.7 g/kg, respectively [26]. The extraction and purification methods are important factors affecting the yield. Table 1 summarizes the extraction yield of the PGG-containing crude extracts and purified PGG obtained from various plants. 

Several methods have been developed to extract and purify PGG from plants, and ethanol [12,27,28,29,30,31,32], methanol [25,26,33,34,35], and aqueous acetone [36] are the commonly used solvents for extraction. However, researchers have not compared the extraction yield of PGG obtained using different extraction methods. Most of them have commonly used ethanol or methanol as an extraction solvent for PGG, whereas a few have used water and aqueous acetone. Notably, in various recent studies, methanol or ethanol was the common extraction solvent. Several purifying methods have been used, such as high-performance liquid chromatography (HPLC) [12], capillary electrophoresis (CE) [29], and Sephadex LH-20 column chromatography [37]. Juang et al. compared the yield of PGG purified using HPLC and CE [29] and found that the extraction yield was higher with CE than with HPLC (plants and extraction methods were the same in both cases). However, CE was used for purifying PGG only in this study, and HPLC was the most commonly used method for purifying PGG.

## 3. Anticancer Activity of PGG

PGG has a cytotoxic effect on many cancers, including prostate, breast, lung, head and neck, liver, leukemia, cervical, colorectal, and pancreatic cancers. Here, we summarized the studies on the anticancer effects of PGG on various cancer cell lines and animal models in the following sections (Table 2 and Table 3). PGG can affect different cancer stages and inhibit tumor growth through multiple mechanisms depending on cell origin, with minimal toxicity against normal cells. PGG targets several aberrant signal-transduction pathways that control cell growth and division, apoptosis, angiogenesis, and metastasis.

### 3.1. Breast Cancer

PGG has exhibited anticancer properties in various breast cancer cell lines, including the triple-negative breast cancer cell lines (such as MDA-MB-231 and MDA-MB-468) and the estrogen receptor-positive breast cancer MCF-7 cell line. Mendonca et al. [38] found that PGG inhibited tumor necrosis factor (TNF)-α-activated CXCL1/GRO-α expression by inhibiting nuclear factor kappa-light-chain-enhancer of activated B cells (NF-κB) and mitogen-activated protein kinase (MAPK) signaling pathways in triple-negative breast cancer cell lines. The compound inhibited cell proliferation and induced apoptosis. Chai et al. demonstrated that PGG induced G1- and S-phase arrest by decreasing cyclin D1 concentration in vitro [39]. In a mouse animal model, gavage administration of PGG inhibited the MDA-MB-231 xenograft growth by >60%. Chai et al. reported that PGG suppressed triple-negative breast cancer xenograft growth and metastasis by inhibiting the JAK1–signal transducer and activator of the transcription (STAT)3 signaling pathway [40]. PGG suppressed the growth of MDA-MB-231 cells by downregulating fatty acid synthase (FAS); this enzyme activates caspase-3 and is highly expressed in some cancers [41]. Deiab et al. found that PGG inhibited the proliferation of MDA-MB-231 cells by inhibiting human lactic acid dehydrogenase-A [42]. Kantapan et al. revealed that PGG induced apoptosis in MCF-7 breast cancer cells by increasing reactive oxygen species (ROS) production, promoting mitochondrial membrane depolarization, and increasing the Bax/Bcl-2 ratio, indicating that PGG induced apoptosis in cancer cells by activating mitochondria-mediated pathway [43].

### 3.2. Prostate Cancer

Prostate cancer is the second leading cause of cancer-related deaths among men. Several authors have suggested the potential use of PGG as a chemopreventive agent against prostate cancer. PGG inhibits the growth and proliferation of prostate cancer cells by targeting multiple molecular pathways. For instance, PGG hindered the growth of prostate cancer cells by inhibiting epidermal growth factor (EGF)-induced nuclear translocation of NF-κB and subsequent activation of c-Jun N-terminal kinase (JNK), an upstream modulator of NF-κB [44]. Further, the authors intratibially injected PC-3 prostate cancer cells into nude mice, followed by an intraperitoneal injection of PGG, and found that PGG suppressed tumorigenesis in these mice. Hu and colleagues suggested that PGG activated the caspase-mediated apoptosis in DU145 and LNCaP prostate cancer cells to exert its anticancer effect. Notably, these two cell lines differed in p53 functionality. The apoptotic effects induced by PGG in the p53-mutant prostate cancer DU145 cells were linked to the inhibition of STAT3 phosphorylation followed by the downregulation of STAT3 transcriptional target genes Bcl-XL and Mcl-1. In contrast, the apoptosis of p53 wild-type LNCaP cells was mediated by PGG-induced ROS production that activated p53 [45]. PGG also induced autophagic cell death in prostate cancer cells with an aggressive phenotype (PC-3 cells with caspase-resistant properties) [46]. PGG induced cell cycle arrest by affecting DNA replication and reducing the expression of cyclin D1 [47]. Taken together, PGG acts on multiple targets and can be further developed as a potential candidate for prostate cancer therapy.

### 3.3. Lung Cancer

Angiogenesis is the formation of new capillaries from existing blood vessels in tumors for growth, invasion, and metastasis. Huh et al. revealed that PGG inhibited the growth of MRC5-SV2 lung cancer cells by inhibiting cyclooxygenase-2 and MAPK-dependent signaling pathways, which, in turn, inhibited angiogenesis [48]. The result showed that PGG treatment notably decreased tumor volume over time, and tumor weight decreased to 43% and 9% of that in the control group in low- and high-dose groups, respectively.

### 3.4. Liver Cancer

PGG showed promising therapeutic potential in hepatocellular carcinoma (HCC). Oh et al. determined that PGG inhibited the growth of SK-HEP-1 cells (an HCC cell line) by arresting the cell cycle in the G0/G1 phase and inhibiting the activation of NF-κB [49]. Yin et al. demonstrated that PGG induces senescence-like S-phase arrest in hepatoma cell lines (HepG2 and Huh-7) by increasing intracellular ROS production [50]. Moreover, PGG induced autophagy-mediated senescence-like arrest in liver cancer cells [51]. Recently, Kant et al. screened a natural compound, PGG, which worked as a glycine N-methyltransferase (GNMT)-inducer in hepatocellular carcinoma (HCC) therapy [52]. GNMT is a tumor suppressor for HCC because it protects the cells from the cytotoxicity induced by carcinogens. Notably, GNMT was downregulated in the tumor tissues collected from patients with HCC. The authors also evaluated the antiproliferative effect of PGG on multiple HCC cell lines, including Huh7, Hep 3B, SK-HEP-1, Mahlavu, and HepG2. They found that PGG inhibited the proliferation of HCC cells in a dose-dependent manner. Further, PGG inhibits the growth of Huh7 xenograft tumors in a mouse model by inducing the expression of GNMT. In another study, the same group of authors revealed that PGG induced GNMT through proteasome-independent MYC downregulation [53]. These findings indicated that PGG induced GNMT to exert its antiproliferative effect on HCC cells. Therefore, PGG shows notable therapeutic potential for liver cancers.

### 3.5. Pancreatic Cancer

PGG acted as an insulin-mimetic compound that damaged pancreatic cancer cells (MiaPaCa2 and Panic-1) and alleviated cachexia in tumor-bearing mice by inhibiting insulin receptor/insulin-like growth factor receptor-1 activity and decreasing glycolytic enzymes in pancreatic cancer cells [54]. The cluster of differentiation (CD)44 is a critical cancer stem cell (CSC) marker associated with pancreatic cancer, and pancreatic CSCs are vital in sustaining continuous tumor growth and chemoresistance [55,56]. Patients with CD44-positive pancreatic cancer have shorter median survival than those with CD44-negative disease [57]. Kim et al. revealed that PGG inhibited the expression of pancreatic CSCs, CD44, and CD44v3 by inducing the phosphorylation of p53 and suppressing NF-κB and fork-head box O3. This resulted in the downregulation of CSC regulatory factors, namely Nanog, Oct-4, and Sox-2, which act downstream of CD44v3 signaling. These findings suggested that PGG can inhibit CSC markers and may have a therapeutic effect on pancreatic cancer [58].

### 3.6. Head and Neck Cancer

Kantapan et al. found that PGG extracted from the Bouea macrophylla seeds inhibited the growth of human head and neck squamous cell carcinoma CAL27 and FaDu cell lines [12]. Recently, Fan and colleagues tested the anticancer effect of PGG on nasopharyngeal cancer cells (CNE1 and CNE2) and found that it regulated the cell cycle by affecting the expression of p53, cyclin D1, cyclin-dependent kinase (CDK)2, and cyclin E1 proteins. Moreover, PGG induced apoptosis and autophagy in these cell lines. In addition, PGG decreased NPC cell migration by increasing E-cadherin and decreasing N-cadherin, vimentin, and CD44 protein concentrations, thereby downregulating the p-mTOR and β-catenin expression. Overall, PGG inhibited nasopharyngeal cancer cell growth and lung metastasis [59].

### 3.7. Colorectal Cancer

Colorectal cancer is the third most common cancer and ranks second in cancer-related mortalities [1]. PGG suppressed the growth and proliferation of colorectal cancer cells. The researchers treated HCT116 and HT29 colorectal cancer cells with different concentrations of PGG for 48 h, and the corresponding IC50 values were displayed in Table 2 [60]. PGG induced the expression of p53 while increasing the expression of p21. PGG affected the expression of cell cycle-related proteins (such as cyclin E and CDK2) and inhibited apoptosis-related proteins (Bcl-2 and cleaved caspase-3).

### 3.8. Glioma Cancer

Glioma cancer is a common intracranial tumor. PGG inhibits glioma cancer cells by suppressing fatty acid synthase and activating caspase-3 [41]. The IC50 of PGG for glioma cancer cells treated for 24 h was 25 µM.

### 3.9. Cervical Cancer

Vaccinia H1-related phosphatase (VHR) dephosphorylates MAPKs, such as extracellular signal-regulated kinase (ERK) and JNK [61]. VHR is upregulated in various cervical cancer cell lines [62]. PGG inhibited the catalytic activity of VHR in vitro. The incubation of HeLa cervical cancer cells with PGG markedly decreased their viability, reduced the concentration of cyclin D1 and Bcl-2, and inhibited STAT3 phosphorylation [63].

### 3.10. Leukemia

Leukemia is a systemic cancer commonly treated using chemotherapy. Acquired drug resistance is a common complication of the available therapeutic options, and patients eventually develop relapsed or refractory disease [64]. Moreover, chemotherapeutic drugs used for leukemia treatment have high costs and severe side effects [65]. Several authors have reported the antileukemic effects of PGG. Pan et al. showed that PGG effectively inhibited the growth of human promyelocytic leukemia HL-60 cells and induced apoptosis in them [66]. In addition, Kwon et al. demonstrated that PGG enhanced the anticancer activity of imatinib in chronic myelogenous leukemia K562 cells in mice through the ROS-dependent JNK and down-regulated domain-associated protein (DAXX) signaling pathway [67]. Recently, Tseeleesuren and coworkers reported that PGG has therapeutic potential for multiple myeloma. In this study, PGG inhibited MYC transcription and promoted MYC degradation through a proteasome-independent pathway, thereby inducing G1-phase cycle arrest and apoptosis in multiple myeloma cell lines [68].

**Table 2:** In vitro studies on the anticancer activity of PGG.

Cancer Type and Cell Lines	Plant Source of PGG	IC50 (Exposure Time)	Effect	References
**Breast**				
MDA-MB-231		47.25 ± 2.03 µg/mL (24 h)	Inhibit tumor cell proliferation and induce cell apoptosis	[38]
		<11.76 µg/mL (72 h)	Induced cell S-phase arrest	[39]
		23.52 µg/mL (24 h)	Inhibit tumor cell growth	[41]
	Gallnut of *Rhus* *chinensis* Mill	1.13 µg/mL (72 h)	Attenuate cell proliferation	[42]
	*Bouea macrophylla* seeds	26.46 ± 6.53 µg/mL (72 h)	Induce cell apoptosis	[43]
MDA-MB-468		33.60 ± 0.70 µg/mL (24 h)	Inhibit tumor cell proliferation and induce cell apoptosis	[38]
MCF-7		<11.76 µg/mL (72 h)	Induced cell S-phase arrest	[39]
	*Bouea macrophylla* seeds	>100 µg/mL (72 h)	Induce apoptosis	[43]
**Lung**				
MRC5-SV2	*Anacardium occidentale* L.	52.24 µg/mL (48 h)	Induce cell oxidative stress, cytotoxicity	[69]
LLC	Gallnut of *Rhus chinensis* Mill	>70.55 µg/mL(48 h)	Induce cell apoptosis	[48]
**Liver**				
Huh7	*Paeonia lactiflora*	30 µg/mL (72 h)	Induce cell apoptosis, reduced the colony formation	[52]
Hep G2	*Paeonia lactiflora*	160 µg/mL (72 h)	Inhibit tumor cell proliferation	[52]
Hep3B	*Paeonia lactiflora*	70 µg/mL (72 h)	Inhibit tumor cell proliferation	[52]
SK-HEP-1	*Paeonia lactiflora*	100 µg/mL (72 h)	Inhibit tumor cell proliferation	[52]
HepG2		27.94 µg/mL (48 h)	inhibit the proliferation, migration and invasion, induce G1 arrest and apoptosis	[70]
**Prostate**				
LNCaP	Gallnut of *Rhus chinensis* Mill	≤23.52 µg/mL (96 h)	Induce G1-cell cycle arrests	[47]
DU145		≤23.52 µg/mL (96 h)	Induce S-cell cycle arrests	
**Head and Neck**				
CAL27	*Bouea macrophylla* seed	16.68 ± 1.20 µg/mL (48 h)	suppress the tumer cells stemness trait	[12]
FaDu	*Bouea macrophylla* seed	26.50 ± 1.46 µg/mL (48 h)	suppress the tumer cells stemness trait	[12]
**Colorectal**				
HCT116		0.65 ± 0.34 µg/mL (48 h)	Induce cell apoptosis	[60]
HT29		4.19 ± 1.09 µg/mL (48 h)	Induce cell apoptosis	[60]
**Glioma cancer**				
U87		23.52 µg/mL	Inhibit tumor cell growth	[41]

LLC, Lewis lung carcinoma.

**Table 3:** In vivo studies on the anticancer activity of PGG.

Cancer Type and Cell Lines	Plant Source of PGG	Model Treatment Dose	(Administration Route)	Eeffect	References
**Breast**					
MDA-MB-231		MDA-MB-231 injected subcutaneously into the right flank of each 6-week-old female athymic nude mouse	20 mg/kg (O.G.)	Inhibition of breast cancer cell growth	[39]
	Gallnut of *Rhus chinensis* Mill	MDA-MB-231 injected subcutaneously into the right flank of a 6-week-old female BALB/c athymic nude mice	10 mg/kg (O.G.)	Inhibition of MDA-MB-231 xenograft growth and lung metastasis	[40]
**Lung**					
LLC	Gallnut of *Rhus chinensis* Mill	tumor inoculation in C57BL/6 mice	4 or 20 mg/kg (I.P.) alternate days for 17 days from day 3	Decrease in tumor volume over time, suppression of the weight of the tumor, and inhibite tumor angiogenesis	[48]
**Liver**					
Huh7	*Paeonia lactiflora*	Huh7 cells subcutaneously implanted subcutaneously into Balb/c nude mice	300 mg/kg (O.G.)	Suppression of the tumor growth by inhibiting the expression of MYC	[52,53]
**Prostate**					
PC-3		Intratibial injection of PC-3 in nude mice	25 mg/kg (I.P.)	Suppression of Tumorigenesis in nude mice	[44]
**Pancreatic**					
MiaPaCa2		tumor cells were transplanted subcutaneously in male athymic Balb/c mice	20 mg/kg (O.G.)	Alleviates cancer cachexia	[54]
		A piece of tumor tissue made of MiaPaCa2 cells was embedded orthotopically in athymic mice	20 mg/kg (O.G.)	Intra-pancreatic insulin normally combated the pharmacologic effects of PGG	[71]

O.G., Oral gavage; I.P., Intraperitoneal injection.

## 4. Molecular Mechanisms of Anticancer Effects of PGG

Researchers have reported that PGG can act on multiple targets and suppress several aberrant signaling pathways in cancer cells. The mechanisms by which PGG exerts its anticancer effects include an increase in oxidative stress, arresting the proliferation of abnormally growing cells at cell cycle checkpoints, enhanced autophagy and apoptosis of cancer cells, and inhibition of angiogenesis and metastasis. The potential therapeutic targets of PGG are transcription factors, namely STAT3 [40,45,61] and NF-κB [24,39,44,72,73,74], and growth factors, including vascular endothelial growth factor (VEGF; the primary target) [48,75,76,77]. PGG has anti-inflammatory activity, and its principal therapeutic targets are TNF-a [38,78,79,80,81,82,83], interferons [74,83,84,85], interleukins [78,80,82,83,84,85,86], and MCP-1 [79,80,87]. PGG also targets many apoptotic proteins, such as poly (ADP-ribose) polymerase (PARP) [48,67,86], Bax [43,88,89], and caspase-3 [41,60,67,68,88]. The central main protein kinases targeted by PGG are MAPK [24,38,44,73,80,82,90], ERK [91,92,93], JNK [40,51,67,94], and PI3K/AKT [70,95], and the major cell survival/proliferation proteins targeted by PGG are Bcl-2 [60,70,89,96] and cyclin D1 [39,47,49,53,61,89].

### 4.1. Effect of PGG on Cell Cycle Arrest

Cell cycle arrest is the process in which the cells temporarily stop their cell cycle progression to facilitate DNA repair before continuing cell proliferation. This process is irreversible and can ultimately result in apoptotic cell death if the damage cannot be repaired [97]. CDKs are a critical component of the cell cycle machinery that drives phase transition in the cell cycle. They tightly regulate the cell cycle progression by forming a complex with their respective cyclin proteins. CDK activity is negatively regulated by CDK inhibitors [98]. Several phytochemicals exert their antiproliferative effect by modulating the genes that control several aspects of the cell cycle (such as cyclins and CDKs). Notably, these genes are abnormally expressed in most cancer cells [99,100]. PGG targets CDK and its inhibitor to suppress tumor progression. Recently, Kawk and coworkers revealed that PGG directly targeted the deregulation of CDK2 and its regulatory cyclin (cyclin E). PGG decreased CDK2 and cyclin E expression and caused G1 cell cycle arrest, concomitant with the inhibition of HCT116 and HT29 colon cancer cell proliferation. In addition, PGG promoted the expression of several CDK inhibitors, such as p21 [60]. Chen et al. reported that PGG induced G1-phase cell cycle arrest in breast cancer MCF-7 cells. The molecular mechanism involved an increase in the expression of CDK inhibitors of the Cip/Kip family, including p21Cip and p27Kip proteins, and a decrease in the activity of cyclin D/CDK4 and cyclin E/CDK2 [101]. Notably, a combination treatment of PGG and 5-fluorouracil markedly induced G1-phase cell cycle arrest in HepG2 liver cancer cells. Western blot analysis revealed that an increase in the p27 expression and a decrease in cyclin E1 was an essential step in the G1-phase cell cycle arrest, thereby inhibiting cell proliferation [70]. Therefore, PGG acts as a cell cycle modulatory agent in CDK-overexpressing cancer cells. The proto-oncogene, cyclin D1, is a critical regulator that drives the progression of a cell from the G1 to the S phase of the cell cycle. Cyclin D1 forms a complex with its binding partners, namely CDK4 and CDK6, and then promotes cell cycle progression by inhibiting the activity of the retinoblastoma protein [102,103]. The altered regulation of cyclin D1 has been implicated in the progression of various cancers, including breast, esophagus, bladder, and lung cancers [104,105,106,107]. Several authors have reported that PGG treatment downregulated cyclin D1 in several cancer cell lines, thereby leading to tumor suppression. For instance, a lower dose of PGG induced the S-phase arrest, whereas a higher dose induced the G1-phase arrest without inducing p21Cip1 and p27Kip1 expressions in DU145 prostate cancer cells. Apparently, PGG induced the S-phase arrest by blocking DNA replication, and the G1-phase arrest was induced by downregulating cyclin D1 [47]. Similarly, PGG induced S-phase arrest in triple-negative breast cancer cells without inducing p21Cip1 and p27Kip1 expressions. The molecular mechanism analysis suggested that PGG induced S-phase arrest by inhibiting DNA replication and induced G1-phase arrest by reducing the expression of cyclin D1, which, in turn, inhibited tumor growth in a triple-negative breast cancer xenograft model [39]. PGG decreased cell proliferation by inducing cell cycle arrest in hormone receptor-positive (ER+) breast cancer cells. Flow cytometry analysis revealed that PGG blocked the cell cycle at the S phase at a lower dose and the G1 phase at a higher dose. The possible underlying molecular mechanism involved the cyclin D1 downregulation by inhibiting the expression of hepatoma upregulated protein, which is crucial for tumor proliferation, invasion, and metastasis [89]. Yoon et al. found that PGG induced the G1-phase cell cycle arrest in HeLa cervical cancer cells concomitant with markedly decreased cyclin D1 concentrations, which correlated with the inhibition of the catalytic activity of VHR [63]. Overall, PGG may exert its anticancer activity by modulating the cell cycle in cancer cells.

### 4.2. Effect of PGG on Inducing Cell Death (Apoptosis and Autophagy)

Apoptosis is a programmed cell death vital to maintain cell homeostasis. Apoptosis induction is a critical target in many cancer treatment strategies [108]. Cell death is determined by the balance between antiapoptotic Bcl-2 family proteins (such as Bcl-2 and Bcl-XL) and pro-apoptotic Bcl-2 family proteins (such as Bax and Bak) [109]. PGG can induce various types of cell death, including apoptosis and autophagy. Xiang et al. showed that PGG affected the expression of Bcl-2 and Bax and downregulated the Bcl-2/Bax ratio in a dose-dependent manner in anti-ER-positive breast cancer cells [89]. PGG induced the apoptosis of colon cancer cells by reducing the expression of Bcl-2 [60]. Therefore, PGG induced apoptotic cell death by modulating the expression of the pro-apoptotic Bcl-2 family proteins. An excessive generation of ROS is cytotoxic and leads to apoptosis induction in cancer cells [67,88]. ROS are critical in activating the apoptosis signal-regulating kinase 1/MAPK signaling pathway [110]. Several authors have elaborated on the antioxidant activity of PGG. However, some authors have also reported that PGG increases ROS production. Kwon et al. showed that PGG markedly enhanced the generation of ROS in chronic myeloid leukemia K562 cells, mediated by the activation of JNK and DAXX [67]. Hu et al. showed that PGG-induced ROS generation mediated p53 activation and apoptosis in LNCaP cells [45]. Yin et al. demonstrated that PGG-induced intracellular ROS generation induced a senescence-like response in human hepatoma and breast cancer cells [50]. Overall, these studies suggested that PGG induced ROS generation and, subsequently, apoptosis to exert its anticancer activity. Recently, Mendonca et al. demonstrated a novel mechanism by which PGG induces cell death [38]. Several death receptors of the TNF receptor superfamily, including death receptor (DR)4, DR5, DR6, and CD137, were upregulated in the triple-negative breast cancer cells treated with PGG. These receptors can activate the extrinsic apoptosis pathway irrespective of the p53 activation or suppression. These findings suggested that PGG can treat cancers where p53 signaling is not activated [38]. 

PC-3, an aggressive prostate cancer cell line, lacks p53 expression but has a high basal expression of AKT, which may contribute to its resistance to caspase-mediated apoptosis. PGG-induced autophagy in PC3 cells. The microtubule-associated protein 1 light chain 3, a biochemical marker for autophagy, was upregulated in these cells, and PGG-induced autophagy was associated with the inactivation of mTOR downstream targets [46]. 

### 4.3. Effect of PGG on the Inhibition of Angiogenesis and Metastasis

Tumor angiogenesis is a hallmark of tumor progression and essential for solid tumors’ growth, survival, and metastasis [111]. Tumor angiogenesis is the proliferation of new blood vessels to procure nutrients and oxygen essential for tumor growth. Tumor cells secrete critical molecules called pro-angiogenic factors, such as VEGF and matrix metalloproteinase (MMP)-9, to drive tumor-induced neovascularization [112]. PGG modulates tumor growth by inhibiting several pro-angiogenic stimulators. It inhibits VEGF-induced human umbilical vein endothelial cell proliferation and the growth of immortalized human microvascular endothelial cells by inhibiting the binding of VEGF to its receptor [75]. Park et al. found that PGG suppressed VEGF secretion in hypoxia-inducible prostate cancer LNCaP cells and inhibited capillary tube formation in human umbilical vein endothelial cells maintained in a conditioned medium. Hypoxia is a critical factor for inducing the transcription of VEGF by modulating the hypoxia-inducible factor-1 (HIF-1) concentration [113]. PGG suppressed angiogenesis by inhibiting HIF-1, thereby suggesting that PGG has antiangiogenesis and chemopreventive activities in prostate cancer cells [11]. STAT3, a cytoplasmic transcription factor, is critical in inflammation and immune system regulation. Cancer cells with activated STAT3 show angiogenesis, inflammation, and metastasis [114]. VEGF is one of the well-recognized target genes of STAT3 [115]. Oral PGG treatment of MDA-MB-231 xenograft nude mice suppressed phosphorylated STAT3 in the tumor tissues and downstream target proteins, including VEGF and Bcl-2, which explained the antiproliferative, pro-apoptotic and antiangiogenesis effects of PGG in a breast cancer mouse model [40]. Capillary morphogenesis gene 2 (CMG2) is a transmembrane extracellular matrix-binding protein involved in cell adhesion, migration, and survival. High-affinity CMG2 binders can inhibit angiogenesis and tumor growth, and PGG is an inhibitor of CMG2 with antiangiogenic activity [116]. Taken together, PGG inhibits angiogenesis in several types of cancers by suppressing the expression of angiogenesis-stimulating factors, such as VEGF, CMG2, and EGF.

The final event in carcinogenesis and a critical hallmark of cancer is tumor metastasis. It is a complex cascade involving an active migration of aggressive tumor cells from their origin into nearby or distinct organs [117]. Metastasis develops because of specific alterations in tumor cells, including the degradation of the extracellular matrix by MMPs and an aberrant expression of the epithelial-to-mesenchymal transition (EMT)-translational factors. EMT is a process by which cells lose their epithelial characteristics and acquire mesenchymal traits concomitant with the changes in the expression of cell adhesion molecules. EMT enhances invasion to confer metastatic properties to tumor cells [118]. In addition, tumor inflammation and angiogenesis are critical for the metastasis cascade [119]. Therefore, inhibition of tumor migration is an important therapeutic approach in metastatic tumor therapy. PGG has the potential to treat metastatic tumors. Ho et al. showed that PGG inhibited the migration of melanoma cancer cells in vitro by inhibiting the activity of MMP-9 (a metastatic marker) by suppressing the activation of transcription factor AP-1 [120]. Moreover, Kuo and colleagues showed that PGG treatment decreased the MMP-9 activity in prostate cancer cells and inhibited bone metastasis by blocking the EGF-induced JNK1/2 and NF-κB signaling pathways [44]. The finding was crucial because bone metastasis is observed in more than 50% of the patients with prostate cancer [121]. Bone-derived EGF may contribute to prostate cancer metastasis [122]. Lin et al. suggested that PGG reduced the expression of EGF by inhibiting the PI3K/AKT/mTOR signaling pathway [96]. Recently, Yang et al. reported a new mechanism by which PGG inhibited metastasis of colorectal cancer cells [123]. PGG treatment inhibited cell adhesion, motility, and migration by regulating the expression of cathepsin B in colorectal HCT116 and colon 26-M01 cancer cells. Notably, cathepsin B (lysosomal cysteine protease) is a critical matrix protease involved in tumor metastasis [124]. Further, modulation of the cathepsin B expression contributed to the inhibition of EMT. Overall, PGG showed a promising antimetastatic effect in colorectal cancer by regulating cathepsin B-mediated extracellular matrix dynamics and EMT [123]. Fan et al. recently demonstrated that PGG inhibited the EMT and cell migration in nasopharyngeal cancer cells by altering the EMT-translational factors (including β-catenin, cyclin D1, CD44, and E-cadherin) and suppressing the Wnt/β-catenin pathway. Moreover, PGG treatment decreased tumor volume and reduced lung metastasis in a CNE2 xenograft mouse model. [59]. Overall, PGG is a potent antiangiogenesis and antimetastatic agent that can be used in cancer therapies.

### 4.4. Effect of PGG on STAT3 Transcription Factors

Transcription factors are crucial for tightly regulating gene expression in diverse biological processes. Dysregulation of several transcription factors contributes to carcinogenic processes, including cancer development, cell survival, cell proliferation, and tumor angiogenesis [125]. STAT is a cytoplasmic transcription factor that mediates cellular responses to different cytokines and growth factors. STAT activation is crucial in inflammation and cancer. Among the STAT family members, STAT3 and STAT5 are involved in cancer progression (especially STAT3) [126]. The constitutive activation of STAT3 plays a vital role in tumor formation, development, metastasis, and recurrence, and inhibition of STAT3 activation leads to tumor growth repression in several cancer cell lines [127]. Therefore, several treatment strategies target the STAT3 pathway for cancer therapy. PGG suppresses the STAT3 expression and activation in various cancer cells, including prostate, breast, head and neck, and cervical cancer cells. PGG inhibited STAT3 Tyr705 phosphorylation in the prostate cancer DU145 cell line, thereby downregulating STAT3 transcriptional targets, such as Bcl-XL and Mcl-1, which decreased cell viability and increased caspase-mediated apoptosis. Moreover, PGG markedly inhibited the growth of DU145 prostate cancer tumor xenografts in a mouse model concomitant with the inhibition of phosphorylated STAT3 [45]. Similarly, Lee and coworkers demonstrated that oral gavage treatment of PGG inhibited triple-negative breast cancer growth and metastasis by downregulating pSTAT3 and its downstream target proteins and subsequent caspase activation. However, pervanadate, a phosphatase inhibitor, reversed the effect of PGG-induced downregulation of pSTAT3 and caspase activation [40]. Our research group recently revealed the antiproliferative effect of PGG on head and neck squamous cell carcinoma cells. The results of in silico molecular docking and western blotting suggested that PGG treatment reduced the expression of phosphorylated STAT3. The computational approaches indicated that PGG was an inhibitor of STAT3 [128]. In addition, PGG suppressed the STAT3 expression in HeLa cervical cancer cells, thereby decreasing cell viability and increasing the cleaved PARP concentrations [63]. Overall, PGG inhibited STAT3 activation from exerting its anticancer effects.

**Figure 2:**
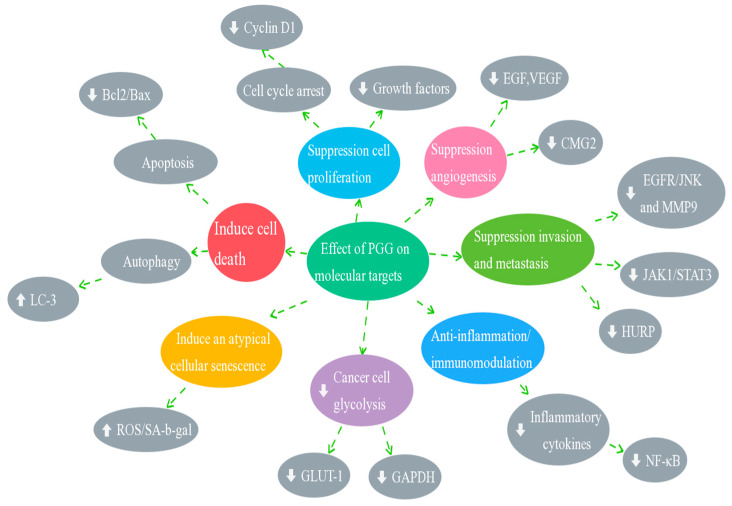
Anticancer effect of PGG on different molecular targets (EGF, epidermal growth factor; VEGF, vascular endothelial growth factor; CMG2, capillary morphogenesis gene 2; JNK, c-Jun N-terminal kinase; MMP-9, matrix metalloproteinase-9; HURP, hepatoma upregulated protein; NF-κB, nuclear factor kappa-B; GAPDH, glyceralde-hyde-3-phosphate dehydrogenase; GLUT1, glucose transporter-1; ROS/SA-gal, reactive oxygen species/senescence-associated galactosidase; LC3, light chain 3. ⇩ Inhibition or downregulation. ⇧ Activate or upregulation).

Table 4 summarizes the molecular targets and signaling pathways in various cancers, and Figure 2 presents the effects of PGG on several molecular targets. PGG targets multiple molecules and signaling pathways in a tumor and the same molecules in different tumors, thereby suggesting that it is a potent antitumor agent.

## 5. Synergistic Effect of PGG on Cancer Chemotherapy and Radiotherapy

Heterogenous tumors consist of cancer cells containing multiple aberrant signaling pathways that fail to respond entirely to conventional therapies, ultimately leading to therapy resistance and tumor relapse in many patients. Therefore, combined treatments with diverse action mechanisms can be more effective against cancer cells than traditional therapies. PGG acts on multiple targets; therefore, it can be combined with classical chemotherapeutics to avoid or minimize the risk of multidrug resistance. The available chemotherapeutic drugs cannot treat advanced multidrug-resistant cancers [131]. The complex mechanisms of drug resistance include overexpression of the ATP-binding cassette transporters (which help efflux certain chemotherapeutics) [132], repair of DNA damage, blocking drug-induced apoptosis, and use of existing detoxifying systems [133]. Radioresistance is also a complex biological process associated with DNA damage, cell cycle arrest, apoptosis, autophagy, gene mutations, and abnormal cell cycle checkpoints [134,135].

Therefore, new tumor therapy strategies aim to enhance tumor sensitivity to primary chemotherapeutic agents and ionizing radiations. Some authors have reported that PGG treatment increased the efficacy of chemotherapy and radiotherapy.

### 5.1. Chemotherapy

Tae-Rin Kwon et al. attempted to treat chronic myelogenous leukemia with a combination of PGG and imatinib [136]. They found that PGG synergistically enhanced the antitumor activity of imatinib in myelogenous leukemia cells. Ding et al. found that a combination of PGG and 5-fluorouracil slowed the growth of aggressive phenotypes of HepG2 cells, increased the Bax/Bcl-2 ratio, activated caspase-9 and caspase-3, and induced apoptosis [70]. This combination treatment increased p27 and cyclin B1 and decreased cyclin E1 concentrations, leading to the G1-phase arrest and downregulation of multidrug resistance 1 and lipoprotein receptor-related protein 1. These findings suggested the potential mechanism by which PGG increased the sensitivity of 5-fluorouracil against aggressive phenotypes of HepG2 cells. Ryu et al. treated the renal cancer cells with a combination of PGG and cisplatin and found that PGG synergistically enhanced cisplatin-induced cell death in a time-dependent manner [88]. The combination index value of PGG and cisplatin at 72 h was 0.204, thereby confirming the synergism of PGG and cisplatin. PGG potentiated the cytotoxicity of cis-platin in both 2D and 3D spheroid cultures of head and neck squamous cell carcinoma cells by promoting apoptosis. The combination treatment was more effective in decreasing the size of 3D multicellular spheroid than monotherapies. Taken together, the combination therapy of PGG and cisplatin synergistically decreased cancer cell viability and induced apoptosis in 2D and 3D models [128].

### 5.2. Radiotherapy

Cancer stem cells (CSCs) are crucial in radiation resistance and the recurrence of cancer. Our research group reported that PGG suppressed the stemness trait and increased the radiosensitivity of head and neck squamous cell carcinoma cell lines in vitro by enhancing radiation-induced DNA damage and consequently increasing cell death [12]. Enhancing the sensitization of cancer cells to conventional treatment approaches is an exciting area of research, and PGG is a promising sensitizing agent for adjuvant therapy in cancer treatment. However, further studies are required to ascertain its role in reducing the adverse effects of conventional treatments on non-cancerous cells and tissues.

## 6. Safety Profile and Pharmacokinetics of PGG

A few studies have reported the safety profile of PGG. Low doses of PGG are considered safe in the diet because it is abundant in fruits and vegetables. Recently, Chen et al. showed that PGG at high doses (100 or 200 mg/kg/day) was orally safe in C57BL/6 mice, and the maximally tolerated dose was up to 200 mg/kg/day. Moreover, these mice did not observe toxic effects and decline in body and organ weights after 7-day PGG treatment. PGG showed low cytotoxicity to three human normal cell lines, including lung epithelial cells (Beas-2B), normal liver hepatocytes (LO2), and human embryonic kidney 293 cells with an IC50 of >100 μM [20]. Ryu et al. showed that PGG protected normal human primary renal epithelial cells from cisplatin-induced cytotoxicity and reduced cisplatin-induced sub-G1 accumulation in these cells [88]. Lin and coworkers showed that <50 µM PGG was not toxic to human epithelial and fibroblast cells [137]. Further, Feldman et al. intravenously administered a 50–60 mg dose of PGG and found a precipitous and lethal drop in blood pressure after 30 min. In contrast, a 30 mg dose had no adverse effect on blood pressure [138]. 

A complete understanding of the pharmacokinetics of PGG is essential for the clinical use of PGG as a natural therapeutic agent. Table 5 summarizes some studies on the pharmacokinetics of PGG [139,140,141]. Currently, the data are available on single-dose intraperitoneal administration of PGG but not on oral administration of the compound. The plasma PGG levels were below the limit of detection (i.e., submicromolar) even when the compound was orally administered to mice at a high dose of 80 mg/kg, thereby indicating the first-pass metabolism for the compound. Jiamboonsri et al. found that the total reclaimed PGG was <2% after oral administration, suggesting that >98% of the compound was metabolized and accumulated in some organs and tissues. The authors also revealed that PGG was primarily eliminated by urine and feces [140]. 

Overall, current data indicate that a high dose of PGG is safe in mice. However, more data are required on the pharmacokinetics and safety profile of PGG in other animal models.

## 7. Conclusions and Future Perspective

Here, we summarized the pleiotropic properties of naturally occurring hydrolyza-ble gallotannin PGG. We found that the natural sources of PGG are abundant; however, the content of PGG is different in different plants. The plants (or their parts) with the highest PGG content were *R. chinensis Mill*, *B. macrophylla seeds*, and *M. indica* kernels. PGG can be commercially obtained in large quantities using available extraction and purification technologies. PGG has many biological activities and can regulate various molecular targets and signaling pathways associated with cancer hallmarks. PGG inhibited the growth in numerous cancer cell lines by targeting PI3K/Akt/mTOR and JNK pathways. Further, PGG inhibited the activation of transcription factor STAT3 as a targeted strategy for cancer therapy. PGG enhanced the sensitivity of the tumors to chemotherapy and radiotherapy, suggesting that PGG is a promising adjuvant in these treatments. Taken together, PGG is an excellent natural phenolic molecule for treating different types of human cancer. We recommend that future studies should focus on determining the pharmacokinetics and safety profile to develop PGG into a clinically useful drug.

## Figures and Tables

**Table 1:** Extraction yield of PGG from various plant sources.

Scientific Name	Common Name	Photo	Plant Part Used	Extraction/Purification Method	Yield		References
					Crude Extract	PGG	
*Bouea macrophylla*	Marian plum, plum mango, Maprang, Gandara, Kundang	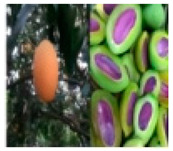	Seed	Ethanol maceration/Cold precipitation followed by centrifugation and HPLC	400 g/kg (MPSE)	52.88 g/kg	[12]
*Mangifera indica*	Mango	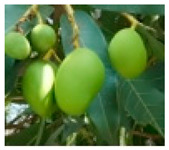	Young leaf	Methanol extraction/HPLC		23.26 g/kg	[26,33]
			Old leaf			1.82 g/kg	
			Peel			17.71 g/kg	
			Kernel			50.03 g/kg	
			Bark			0.7 g/kg	
*Paeonia lactiflora*	Chinese peony, Chinese herbaceous peony, common garden peony	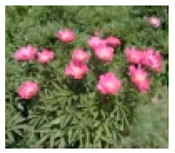	Root	Methanol extraction/HPLC		38.32 g/kg	[34]
*Aloe vera*	Chinese Aloe, Indian Aloe, True Aloe, Barbados Aloe, Burn Aloe, First-aid plant	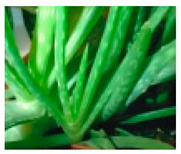	Leaf	Methanol extraction/HPLC		1.520 g/kg	[25]
***Gallnuts****:Galla Chinensis*, *Rhus chinensis*, *Rhus potaninii*, *Rhus punjabensis*, *Quercus infectoria*, *Quercus lusitanica*	Gallnut, nutgall, gall oak, Galla rhois	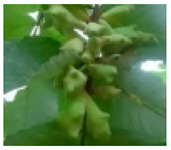	Gall	Methanol extraction/HPLC		58.4 g/kg	[25]
*Scutellaria baicalensis*	Baikal skullcap, Chinese skullcap	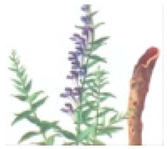	Root	Methanol extraction/HPLC		1.87 g/kg	[25]
*Cassia obtusifolia*, *Cassia tora*	Cassia seed, Semen Cassia	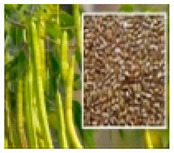	Seed	Methanol extraction/HPLC		1.00 g/kg	[25]
*Radix Paeoniae Alba*	White peony root	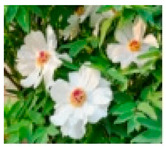	Root	Ethanol extraction/HPLC		2.48 g/kg	[27]
*Radix Paeoniae Rubra*	Red peony root	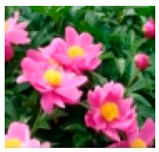	Root	Ethanol extraction/HPLC		2.24 g/kg	[27]
*Terminalia chebula*	Chebulic myrobalan, Black myrobalan, Haritaki	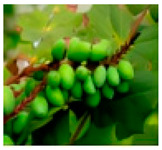	Fruit	Ethanol extraction/HPLC		13.53 g/kg	[28]
				Ethanol extraction/HPLC		14.54 g/kg	[29]
				Ethanol extraction/Capillary electrophoresis		20.7 g/kg	[29]
*Pistacia vera*	Pistachio	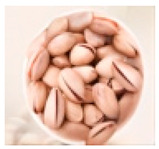	Hull	Methanol extraction/HPLC		9.77 g/kg	[35]
*Elaeocarpus sylvestris*	Woodland elaeocarpus	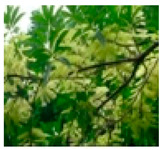	No, specify	Ethanol extraction/Column chromatography/HPLC	200 g/kg	2.4 g/kg	[30]
*Oenothera paradoxa*	Evening primrose	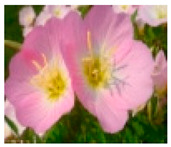	Seed	Ethanolic extraction	745.5 g/kg	16.75 g/kg	[31]
				Ethanolic extraction/HPLC		27 g/kg	[32]

**Table 4:** Mechanism of action of PGG on molecules and pathways involved in various cancer types.

Cancer Type	Mechanism of Action of Pentagalloyl Glucose		References
	Target Pathway/Molecule	Type of Effect	
**Lung cancer**	ERK1/2, pJNK, p38	↓	[48]
	VEGF	↓	
	COX-2	↓	
	p-H2AX	↑	[96]
	CHK2	↑	
	P53	↑	
**Breast cancer**	MAPK	↓	[38]
	IκBKE	↓	
	GRO-α/CXCL1	↓	
	JAK1	↓	[40]
	STAT3	↓	
	HURP	↓	[89]
	Cyclin D1	↓	
	Bcl-2	↓	
	P53Ser15	↑	[39]
	Cyclin D1	↓	
	ROS	↑	[50]
	SA-β-gal	↑	
	LDH-A	↓	[42]
**Liver cancer**	MYC	↓	[53]
	GNMT	↑	
	P27	↑	
	P21	↑	
	Cyclin D1	↓	
	GNMT	↑	[52]
	NF-κB	↓	[50]
	Cyclin D1	↓	
	PI3K	↓	[70]
	Akt	↓	
	Bax	↑	
	Bcl-XL	↓	
	Bcl-2	↓	
	ROS	↓	[50]
	SA-β-gal	↓	
**Brain cancer**	FAS	↓	[41]
	Caspase-3	↑	[41]
**Head and neck cancer**	GSK3β/β-catenin	↑	[59]
	STAT3	↓	[128]
	Bcl-2	↓	
	VEGF	↓	
**Pancreatic cancer**	HIF-1	↓	[54,71]
	Caveolin-1	↓	[54]
	Akt	↓	[54,71]
	ERK	↓	[54,71]
	HK-II	↓	[54,71]
	PFK	↓	[54,71]
	GLUT-1	↓	[54]
	IR/IGF1R	↓	[54,71]
	p-MEK	↓	[71]
	VEGF	↓	[71]
**Colorectal cancer**	GAPDH	↓	[129]
	P53	↑	[60]
	P21	↑	
	Bcl-2	↓	
	c-Caspase-3	↑	
**Prostate Cancer**	P53	↑	[45]
	STAT3	↓	
	pS6K	↓	[46]
	LC3-I/LC3-II	↑	
	PI3K/AKT/mTOR	↓	[95]
	EGF	↓	
	eIF3i	↓	
	PI3K/AKT/mTOR	↓	[11]
	VEGF	↓	
	HIF-1α	¯	
	EGFR/JNK	↓	[44]
	MMP-9	↓	
**Leukemia**			
**-CML**	JNK	↑	[67]
	Mcl-1, Bcl-2, Survivin	↓	
	DAXX	↓	
**-AML**	Bax	↑	[130]
**-MM**	DEPTOR	↓	[68]
	MYC	↓	
**Cervical cancer**	PARP	↑	[63]
	Cyclin D1	↓	
	Bcl-2	↓	
	STAT3	↓¯	

↓ Downregulation/Inhibition; ↑ Upregulation/Activation; p-H2AX, p-H2A histone family member X; CHK2, p-checkpoint kinase 2; IқBKE, I-kappa-B kinase; GRO-α/CXCL1, growth-related oncogene alpha/C-X-C motif ligand 1; JAK1, Janus-activated kinase 1; HURP, hepatoma upregulated protein; ROS, reactive oxygen species; SA-β-gal, senescence-associated β-galactosidase; LDH-A, lactic acid dehydrogenase-A; GNMT, glycine-N-methyl transferase; FAS, fatty acid synthase; HK-II, hexokinase-2; GLUT-1, glucose transporter-1; IR/IGF1R, insulin receptor/insulin-like growth factor-1 receptor; p-MEK, phosphorylated-mitogen-activated protein kinase; GAPDH, glyceraldehyde 3-phosphate dehydrogenase; pS6K, phosphorylation S6 kinase; LC3-I/LC3-II, light chain 3-I/light chain 3-II; PI3K/AKT/mTOR, phosphoinositide-3 kinase/protein kinase B/mammalian target of rapamycin; MMP-9, matrix metalloproteinase-9; DAXX, domain-associated protein; DEPTOR, DEP domain-containing mTOR-interacting protein; GSK3β, glycogen synthase kinase-3 beta.

**Table 5:** Pharmacokinetic parameters of PGG [139,140,141].

Pharmacokinetic Parameter	PGG Dose in the Animal Model		
	Mouse	Rat	
	20 mg/kg (I.P.)	20 mg/kg (I.P.)	20 mg/kg (P.O.)
**Cmax** (μM)	3–4	6.39 ± 4.25	ND
**Tmax** (h)	~2	0.85 ± 0.70	ND
**t_1/2_**(a) (h)	ND		ND
**t_1/2_**(el) (h)	ND	38.66 ± 22.89	ND
**Ke** (h^−1^)	ND	0.023 ± 0.012	ND
**AUC** (0–24 h) (h* µM)	ND	38.78 ± 22.89	ND
**Vd** (L/kg)	ND	7838.89 ± 3474.72	ND
**Cl** (L/h/kg)	ND	30.98 ± 21.73	ND
**MRT** last (h)	ND	12.47 ± 2.77	ND

I.P., intraperitoneal; P.O., per oral (oral administration); ND, not determined.

## Data Availability

No new data were created or analyzed in this study. Data sharing is not applicable to this article.
